# Time-varying mortality risk after gastrointestinal surgery complicated by postoperative infections: a Danish Nationwide study of 859,766 patients

**DOI:** 10.1007/s00423-025-03718-4

**Published:** 2025-05-07

**Authors:** Doruk Orgun, Ask Tybjaerg Nordestgaard, Rasmus Peuliche Vogelsang, Henrik Enghusen Poulsen, Christina Ellervik, Ismail Gogenur

**Affiliations:** 1 Center for Surgical Science, Department of Surgery, Zealand University Hospital Køge and Roskilde, Roskilde, Denmark; 2 Department of Clinical Biochemistry, Copenhagen University Hospital - Herlev and Gentofte, Herlev, Denmark; 3 The Copenhagen General Population Study, Copenhagen University Hospital - Herlev and Gentofte, Herlev, Denmark; 4 Department of Clinical Medicine, Faculty of Health and Medical Sciences, University of Copenhagen, Copenhagen, Denmark; 5 Department of Endocrinology, Copenhagen University Hospital Bispebjerg and Frederiksberg, Copenhagen, Denmark; 6 Department of Cardiology, Copenhagen University Hospital, Hillerød, Denmark; 7 Department of Pathology, Harvard Medical School, Boston, MA USA; 8 Department of Laboratory Medicine, Boston Children’s Hospital, Boston, MA USA; 9 Department of Clinical Biochemistry, Zealand University Hospital, Køge, Denmark; 10 Center for Surgical Science, Department of Surgery, Zealand University Hospital Køge, Lykkebaekvej 1, Køge, 4600 Denmark

**Keywords:** Postoperative infections, Gastrointestinal surgery, Abdominal surgery, Long-term mortality, Time-varying risk, Nationwide study

## Abstract

**Purpose:**

Postoperative infections are associated with increased mortality risk, but it is unclear if this risk increase persists over time. This study aims to estimate mortality risk associated with postoperative infections at different time-points within the first postoperative year in a nationwide cohort of gastrointestinal surgery patients.

**Methods:**

We included all individuals residing in Denmark who underwent gastrointestinal surgery between 1996 and 2018 and were alive at postoperative day 30. For different time-intervals during the one-year follow-up, we calculated mortality rates and cumulative incidences of death for patients with and without 30-day postoperative infections. Time-varying Cox regression analyses estimated the relative mortality risk associated with postoperative infection exposure.

**Results:**

Of 859,766 patients (female:49.2%; median age:51 years), 25,126 (2.9%) had at least one 30-day postoperative infection. In patients with or without infections, cumulative incidences of death between postoperative days 30–365 were 13.5% versus 4.7%. Adjusted hazard ratios (HRs) for mortality from postoperative days 30, 91, 181, and 271 until end of follow-up (until postoperative day 365) were 2.25(95% CI:2.13–2.38), 1.88(1.74–2.04), 1.44(1.29–1.62), and 1.11(1.00–1.28) for any postoperative infection compared to no infection (*p*_time−interaction_<0.001). The adjusted HRs for mortality for postoperative days 30–365 in patients exposed to different postoperative infection types were: sepsis: 4.38(3.90–4.93), pneumonia: 2.60(2.37–2.85), urinary tract infection: 1.26(1.05–1.52), surgical site infection: 1.16(1.04–1.30).

**Conclusion:**

Compared to patients with no infection, patients exposed to 30-day postoperative infections after gastrointestinal surgery had a 2.3-fold risk of mortality at postoperative days 30, and the relative risk of mortality attributed to infection exposure gradually diminished over time.

**Supplementary Information:**

The online version contains supplementary material available at 10.1007/s00423-025-03718-4.

## Introduction

Infections are common postoperative complications that often result in longer hospitalizations, unplanned reoperations, intensive care unit admissions, or death [1–7]. Even following initial recovery and hospital discharge, postoperative infections have been associated with increased mortality beyond the first couple of months after surgery [4–12]. This is particularly true for cancer patients who undergo curative resections, in whom exposure to postoperative infections is associated with an increased risk of cancer recurrence risk and poor overall survival [13–22].

Infections within the first 30 postoperative days have previously been associated with an increased and constant (proportional) risk of secondary infections and mortality within the first year after non-cancer surgery [8]. However, it is unclear whether postoperative infections are associated with an increased long-term mortality risk in a time-dependent fashion where the magnitude of risk changes over time, and whether the mortality risk is different for each postoperative infection type, separately or combined. A more detailed time-dependent postoperative risk profile could help in the planning of future studies of follow-up interventions to increase overall survival among high-risk patients.

In a nationwide cohort of gastrointestinal surgical patients in Denmark between 1996 and 2018, our objective was to first compare absolute risk of mortality for patients with or without 30-day postoperative infections. Then, we investigated the relative risk of mortality associated with 30-day postoperative infections by estimating hazard ratios (HR) for mortality at different time-points within the first postoperative year. We have additionally performed analyses for each major postoperative infection type (sepsis, pneumonia, urinary tract infections (UTI), and/or surgical site infections (SSI)).

## Methods

### Study design

This study is a nationwide registry-based observational study of all individuals who underwent gastrointestinal surgery in Denmark from January 1996 to December 2018. The Danish Data Protection Agency approved access to the Danish Registries (Reference number: 2008-58-0028). According to Danish legislation, registry-based research is exempt from patient consent [23]. Results are reported in compliance with the Strengthening the Reporting of Observational Studies in Epidemiology (STROBE) guidelines.

### Data sources

From the Danish National Patient Registry [24], we collected information on surgical procedures, admission/discharge dates, patient comorbidities and postoperative infections with corresponding diagnosis dates. Date and cause of death information were extracted from the Danish National Causes of Death Registry [25]. Prescription data was collected from the Danish National Prescription Registry [26], which includes all redeemed prescriptions in Denmark since 1995. Demographic data were collected using the Danish Civil Registration System [27]. Data from all registries were combined using the Danish personal identification number (CPR-number), which is unique for every individual.

### Study population

We included all patients who underwent a gastrointestinal surgical procedure that was registered in the Danish National Patient Registry with a Nordic Classification of Surgical Procedures 2005 (NCSP 2005) code: KJ (excluding thoracic esophageal, perianal and purely endoscopic procedures) from January 1st, 1996 to December 31st, 2018. For patients who underwent more than one procedure during the study period, the first recorded non-reoperation procedure was defined as the index surgery. Finally, patients who died in the first 30 days (including the day of surgery) following index surgery were excluded to perform landmark analyses that avoid the introduction of immortal time bias, since the follow-up started at postoperative day 30. This landmark time of 30 days was chosen with the intent of capturing both early and late postoperative infections.

### Postoperative infections

A postoperative infection was defined as at least one hospital-verified diagnosis of infection within the first 30 days following gastrointestinal surgery. The Danish versions of the International Classification of Diseases 10th Revision (ICD-10) codes [28] were used to identify postoperative infections. Infection diagnoses in the Danish National Patient Registry are of high validity, with positive predictive values equal to or above 79% for skin infections, pneumonia, and sepsis [24, 29].

Included postoperative infection types were sepsis/bloodstream infections (referred to as “sepsis”), pneumonia, urinary tract infections (UTI), and surgical-site infections (SSI) (Supplementary Table 1). SSIs included superficial incisional, deep incisional, and organ-space infections (e.g. peritonitis or intraabdominal abscess due to anastomotic leaks).

Exposure groups were either defined as “any postoperative infection” or as each individual infection type (sepsis, pneumonia, UTI, SSI), separately or combined. Patients with more than one hospital-verified infection diagnosis were classified as having “multiple infections”. These included “sepsis combined with pneumonia, UTI, or SSI”, “pneumonia combined with UTI or SSI”, and “UTI combined with SSI”. With this hierarchical classification method, all “multiple infections” categories were exclusive and did not overlap.

### Outcomes

The primary outcome of the study was death that all-cause mortality between postoperative day 30 (start of follow-up) and postoperative day 365. The causes of death are defined by the ICD-10 code that was recorded as the primary cause of death in the Danish Causes of Death Registry [25].

### Co-variables

Co-variables included sex, age, procedure type (gastroduodenal open, gastroduodenal laparoscopic, hepato-pancreato-biliary open, hepato-pancreato-biliary laparoscopic, hernia/abdominal wall open, hernia/abdominal wall laparoscopic, lower GI open, lower GI laparoscopic), elective/emergent surgery, reoperation, year of surgery, perioperative GI cancer diagnosis, hospital region, postoperative length of stay, and patient comorbidities. A reoperation was defined as a procedure coded as “reoperation after gastrointestinal operation” (NCSP 2005 code: KJW) within 30 days after index surgery. Comorbidities were captured using ICD-10 codes from the Danish National Patient Registry and included diabetes mellitus (E10-E14), obstructive pulmonary diseases (J41-J44, J47), ischemic heart disease (I20-I25), heart failure (I50), any cancer history excluding in-situ neoplasms (C00-C99), cerebrovascular disease (I60-I64, G45.9, T81.7Y1), cirrhosis (K70.3, K71.7, K74.3-K74.6), and dialysis-dependent renal failure (N18.5, Z99.2). We further identified patients who had redeemed a prescription of an antidiabetic drug (APC code: A10) to capture diabetic patients treated only in the primary care sector.

### Statistical analyses

All statistical analyses were performed using the R programming language version 4.2.1. The balance of baseline co-variable distributions at the time of surgery according to 30-day postoperative infection status was evaluated by the calculation of standardized mean differences (SMD).

Mortality rates per 1000 person-days and cumulative incidences of death between postoperative days 30–90, 91–180, 181–270, 271–365, and 30–365 (the entire follow-up period) with corresponding 95% confidence intervals (95% CI) were calculated for patients with or without 30-day infection, as well as all individual infection types (sepsis, pneumonia, UTI, SSI) including “multiple infections”. Subsequently, Kaplan-Meier curves for one-year mortality were generated for the same groups. The curves were compared using the log-rank test, and P values less than 0.05 were considered statistically significant.

Finally, multivariable Cox regression models were utilized to estimate hazard ratios (HRs) for mortality associated with any postoperative infection as well as all individual infection types including “multiple infections” with patients who did not have a postoperative infection set as reference. Adjusted hazard ratios were estimated from postoperative days 30, 91, 181 and 271 until end of follow-up (until postoperative day 365). Since the end of the follow-up period was defined as postoperative day 365, calculating hazard ratios for day 365 was not possible due to having no follow-up time left to assess any statistical significance. Therefore, postoperative day 271 was chosen as the final time point of interest. The Cox models were adjusted for age, sex, surgery-specific co-variables (procedure type, elective/emergent surgery, reoperation, year of surgery, perioperative GI cancer diagnosis, hospital region, postoperative length of stay), and all captured comorbidities. Continuous variables included age and postoperative length of stay, while categorical variables included year of surgery, procedure type, and hospital region. Comorbidities were included as binary variables.

Proportional hazards assumption was tested for all predictors by plotting the changes of β-coefficients and scaled Schoenfeld residuals over time [30]. If the trajectory of the β-coefficient was horizontal (i.e. not significantly varying over time), the proportional hazards assumption was considered to be correct. For predictors that violated the proportional hazards assumption, an exposure-time interaction term was added to the Cox models.

### Sensitivity analyses

Due to the inability to distinguish whether infection diagnoses recorded on the day of admission were already present upon admission or developed postoperatively, we performed Cox regression analyses excluding patients with an infection diagnosis on the day of admission. We also performed hospital-stratified Cox regression analyses to adjust for patient clustering by hospital. Additionally, due to the possibility that postoperative length of stay has acted a mediator for the association between 30-day postoperative infection and mortality, we performed additional Cox regression analyses without covariate adjustment for *postoperative length of stay*.

Second, to assess whether the associations between 30-day postoperative infection and mortality were consistent in the patient subgroups of individuals (A) with or without a perioperative GI cancer diagnosis, (B) below and above 65 years of age, and (C) undergoing emergent or non-emergent surgery, we calculated corresponding time-varying HRs for mortality in these subgroups.

Finally, we performed sensitivity Cox regression analyses by utilizing landmark times of 10 and 20 days instead of 30 days to evaluate if the choice of landmark time as 30 days has substantially affected the results. In these analyses, adjusted hazard ratios were estimated in the same manner as the main analyses from the start of follow-up *(postoperative day 10 or 20)*, 91, 181 and 271 until end of follow-up (until postoperative day 365).

**Fig. 1:**
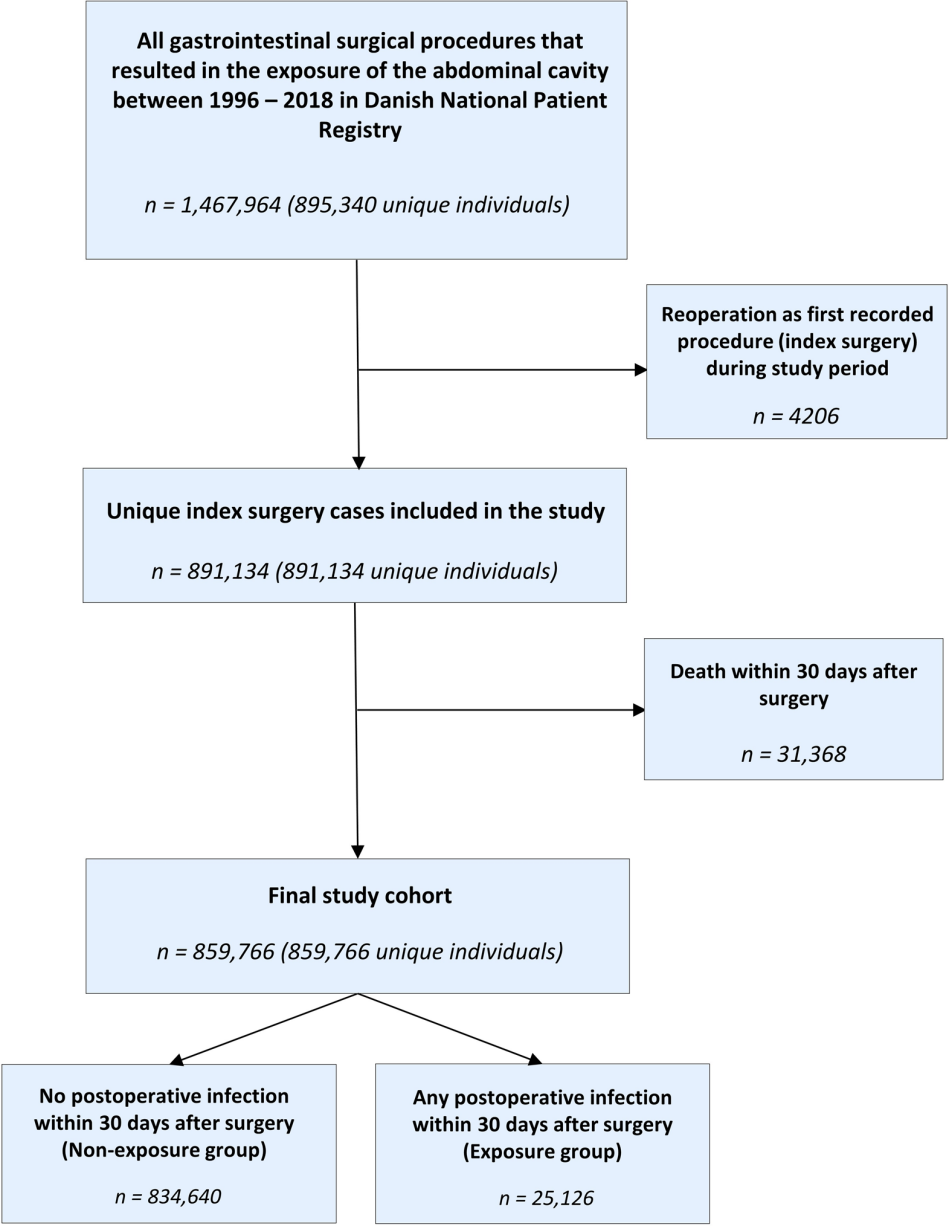
Flowchart of the study inclusion process

**Fig. 2:**
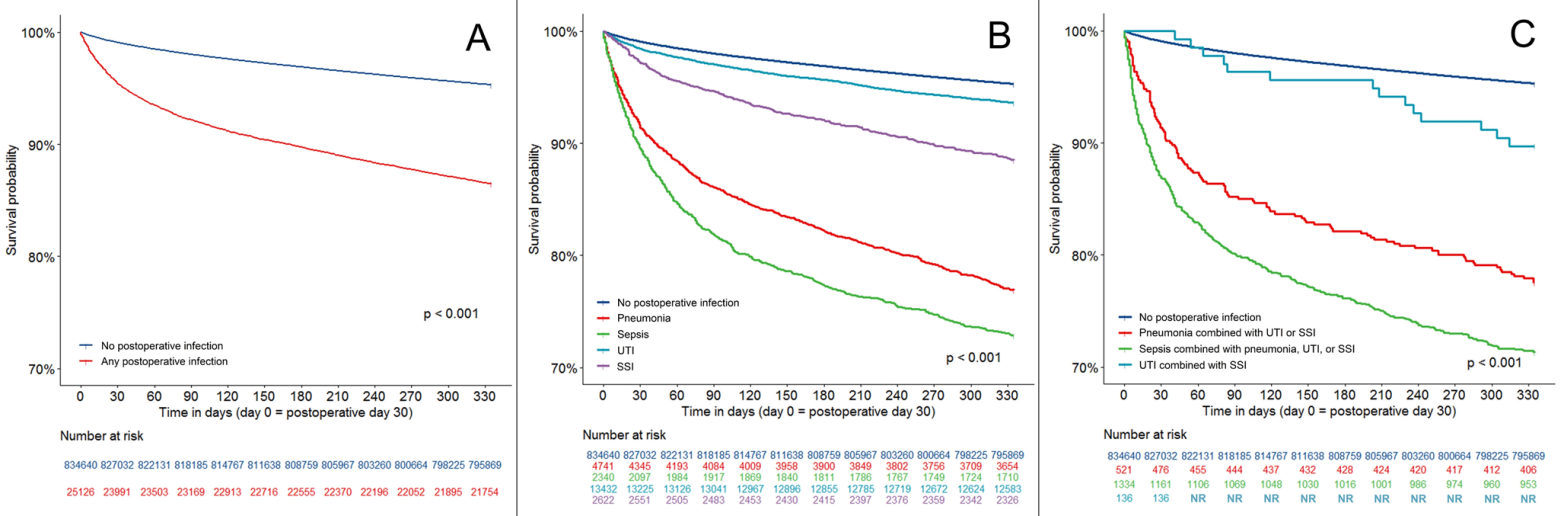
One-year survival Kaplan-Meier curves according to 30-day postoperative infection status. **A**: Kaplan-Meier curves represent survival rates among patients with no postoperative infection (blue curve) and among patients with any type and number of postoperative infections within 30 days postoperatively (red curve). **B**: Kaplan-Meier curves represent corresponding survival rates among patients with no postoperative infection (blue curve) and among patients with one type of infection within 30 days postoperatively by type of infection (light blue, purple, red, and green curves). **C**: Kaplan-Meier curves represent survival rates among patients with no postoperative infection (blue curve) and among patients with multiple infections by type of infection combination (light blue, red, and green curves). [***NR***: *Not reported due to Statistics Denmark regulations; for more information see the Data Availability Statement*]

**Fig. 3:**
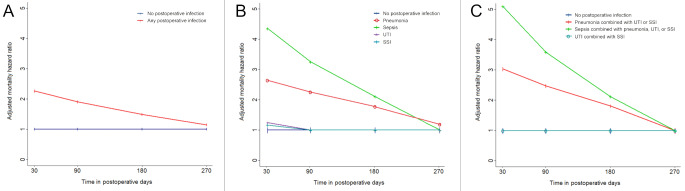
Time-specific multivariable adjusted hazard ratios (HR) for one-year mortality for postoperative days 30–365, 91–365, 181–365, and 271–365 according to 30-day postoperative infection status. Statistically non-significant HR values are shown as HR = 1.00. **A**: HRs for one-year mortality for patients with any type and number of postoperative infections versus patients with no infection within 30 days postoperatively. **B**: HRs for one-year mortality for patients with one type of infection by type of infection. **C**: HRs for one-year mortality for patients with multiple infections by type of infection combination versus patients with no infection within 30 days postoperatively

## Results

We identified 1,467,964 records corresponding to 895,340 unique patients who underwent a gastrointestinal surgical procedure between 1996 and 2018. Subsequently, a total of 891,134 unique index surgery procedures (one procedure per patient) were identified. Then, we excluded 31,368 patients who did not survive the first 30 days following the index surgery, leaving 859,766 patients in the final study (Fig. 1).

**Table 1:** Baseline characteristics of patients who underwent Gastrointestinal surgery in Denmark between 1996 and 2018 according to 30-day postoperative infection status

	No 30-day postoperative infection (*n* = 834,640)	Any 30-day postoperative infection (*n* = 25,126)	Total (*n* = 859,766)	Standardized mean difference
Median age, years [IQR^*^]	50 [32, 66]	60 [41, 72]	51 [32, 66]	0.322
Sex, n (%)				0.023
Female	410,124 (49.1)	12,630 (50.3)	422,754 (49.2)	
Procedure type, n (%)				0.469
Open lower GI ^a^	144,638 (17.3)	7096 (28.2)	151,734 (17.7)	
Lap. ^b^ lower GI	87,117 (10.4)	3490 (13.9)	90,607 (10.5)	
Open upper GI (gastric)	16,341 (2.0)	1023 (4.1)	17,364 (2.0)	
Lap. upper GI (gastric)	22,600 (2.7)	381 (1.5)	22,981 (2.7)	
Open abdominal wall/hernia	355,368 (42.6)	8097 (32.2)	363,465 (42.3)	
Lap. abdominal wall/hernia	55,170 (6.6)	655 (2.6)	55,825 (6.5)	
Open hepato-pancreato-biliary	29,104 (3.5)	2026 (8.1)	31,130 (3.6)	
Lap. hepato-pancreato-biliary	124,302 (14.9)	2358 (9.4)	126,660 (14.7)	
Emergent surgery, n (%)	301,199 (36.1)	14,314 (57.0)	315,513 (36.7)	0.428
Reoperation, n (%)	6843 (0.8)	1967 (7.8)	8810 (1.0)	0.350
Year of surgery, n (%)				0.317
1996–2000	190,575 (22.8)	3052 (12.2)	193,627 (22.5)	
2001–2005	181,103 (21.7)	4818 (19.2)	185,921 (21.6)	
2006–2010	175,283 (21.0)	6160 (24.5)	181,443 (21.1)	
2011–2015	180,266 (21.6)	6951 (27.7)	187,217 (21.8)	
2016–2018	107,413 (12.9)	4145 (16.5)	111,558 (13.0)	
Perioperative GI cancer diagnosis, n (%)	82,866 (9.9)	4409 (17.6)	87,275 (10.2)	0.223
Median length of stay, days [IQR^*^]	1 [0, 4]	3 [1, 8]	1 [0, 4]	0.019
Comorbidities, n (%)				
Diabetes	42,553 (5.1)	2370 (9.4)	44,923 (5.2)	0.168
Cancer history	141,988 (17.0)	7222 (28.7)	149,210 (17.4)	0.282
Coronary artery disease	58,171 (7.0)	3147 (12.5)	61,318 (7.1)	0.188
Heart failure	17,959 (2.2)	1314 (5.2)	19,273 (2.2)	0.164
Dialysis history	1974 (0.2)	203 (0.8)	2177 (0.3)	0.079
Cerebrovascular disease	28,566 (3.4)	1757 (7.0)	30,323 (3.5)	0.161
Obstructive pulmonary disease	29,417 (3.5)	2247 (8.9)	31,664 (3.7)	0.226
Cirrhosis	5327 (0.6)	427 (1.7)	5754 (0.7)	0.099

^*^ IQR: interquartile range

^a^ GI: gastrointestinal

^b^ Lap.: laparoscopic

Descriptive statistics of the patients stratified by infection status (“any 30-day postoperative infection” versus “no 30-day postoperative infection”) are presented in Table 1. The median age of the patients was 51 years (interquartile range (IQR): 32–66) and 49.2% (*n* = 422,754) were female. The median length of hospital stay after surgery was one day (IQR: 0–4), and the most frequent procedures were open hernia/abdominal wall procedures (*n* = 363,465; 42.3%) followed by open lower GI tract procedures (*n* = 151,734; 17.7%). In total, 25,126 (2.9%) were diagnosed with at least one infection within the first 30 days after surgery. Compared with the patients with no postoperative infections, patients with any postoperative infection were older (median age [IQR]: 50 [32–66] versus 60 [41–72]), were more likely to have undergone emergent surgery (36.1% versus 57.0%) or reoperation (0.8% versus 7.8%), had higher prevalence of perioperative GI cancer diagnosis (9.9% versus 17.6%), were hospitalized longer after surgery (median [IQR]: 1 [0–4] day versus 3 [1–8] days), and had higher prevalence of included comorbidities.

Among patients with any postoperative infection, 23,135 had a single infection, and 1991 had at least two types of hospital-verified infections simultaneously (multiple infections). Among the single infection types, SSI was the most frequent with 13,432 cases. The most frequent multiple infection type was “sepsis combined with pneumonia, UTI, or SSI” with 1334 cases (Table 2).

**Table 2:** Cumulative incidences of all-cause mortality and mortality rates in patients who underwent Gastrointestinal surgery in Denmark between 1996 and 2018 according to 30-day postoperative infection status

	Cumulative incidence of all-cause mortality (95% CI ^a^)	Mortality rate per 1000 person-days (95% CI)
No infection (*n* = 834,640)		
Total (postoperative day 30–365)	4.7% (4.7–4.7)	0.14 (0.14–0.15)
Postoperative day 30–90	1.5% (1.5–1.5)	0.25 (0.24–0.25)
Postoperative day 91–180	1.3% (1.2–1.3)	0.14 (0.14–0.14)
Postoperative day 181–270	1.0% (1.0–1.0)	0.11 (0.11–0.11)
Postoperative day 271–365	0.9% (0.9–0.9)	0.10 (0.10–0.10)
Any infection (*n* = 25,126)		
Total (postoperative day 30–365)	13.5% (13.1–14.0)	0.45 (0.43–0.46)
Postoperative day 30–90	6.5% (6.2–6.8)	1.09 (1.04–1.15)
Postoperative day 91–180	3.1% (2.9–3.3)	0.35 (0.32–0.37)
Postoperative day 181–270	2.0% (1.9–2.3)	0.23 (0.21–0.25)
Postoperative day 271–365	1.8% (1.7–2.0)	0.20 (0.18–0.21)
Single infections (postoperative day 30–365)		
Sepsis (*n* = 2340)	27.1% (25.4–29.0)	1.02 (0.94–1.10)
Pneumonia (*n* = 4741)	23.1% (21.9–24.3)	0.82 (0.78–0.87)
Urinary tract infection (UTI) (*n* = 2622)	11.5% (10.4–12.8)	0.37 (0.33–0.41)
Surgical site infection (SSI) (*n* = 13,432)	6.4% (6.0–6.8)	0.20 (0.19–0.21)
Multiple infections (postoperative day 30–365)		
Sepsis combined with pneumonia, UTI, or SSI (*n* = 1334)	28.7% (26.4–31.2)	1.10 (0.99–1.21)
Pneumonia combined with UTI or SSI (*n* = 521)	22.5% (19.1–26.3)	0.80 (0.66–0.96)
UTI combined with SSI (*n* = 136)	10.3% (6.2–16.8)	0.32 (0.18–0.54)

^a^ CI: Confidence interval

5% of the patients (*n* = 42,612) died between postoperative days 30 and 365. Among patients with no postoperative infection, 39 209 (4.7%) died during follow-up and the most common cause of death was cancer-related (69.3% of all deaths) (Supplementary Table 2). Among patients with a postoperative infection, 3403 (13.5%) died during follow-up. In this group, the most common cause of death was also cancer-related (49.2% of all deaths), although these accounted for a lower proportion of all deaths compared to among patients who did not have a postoperative infection (49.2% versus 69.3%; *p* < 0.001) (Supplementary Table 2).

Comparing patients with or without 30-day postoperative infections, mortality rates per 1000 person-days were 0.45 versus 0.14 for the entire follow-up period (postoperative days 30–365), 1.09 versus 0.25 for postoperative days 30–90, 0.35 versus 0.14 for postoperative days 91–180, 0.23 versus 0.11 for postoperative days 181–270, and 0.20 versus 0.10 for postoperative days 271–365. Mortality rates per 1000 person-days were 1.02 for sepsis alone and 1.10 for “sepsis combined with pneumonia, UTI, or SSI” for the entire follow-up period (Table 2).

Corresponding Kaplan-Meier curves for one-year survival for any postoperative infection, any single infection, and any of the “multiple infections” compared with no postoperative infection are shown in Fig. 2A, B and C, respectively. One-year overall survival following any postoperative infection as well as all infection types except for “UTI combined with SSI” were significantly worse compared with no postoperative infections (*p* < 0.001) (Fig. 2A-C).

**Table 3:** Time-specific adjusted hazard ratios (HRs) for mortality for postoperative days 30–365, 91–365, 181–365, and 271–365 in patients who underwent Gastrointestinal surgery in Denmark between 1996 and 2018 according to 30-day postoperative infection status

Adjusted hazard ratios for mortality ^*^ [95% CI ^a^]				
	**Postoperative days 30–365**	**Postoperative days 91–365**	**Postoperative days 181–365**	**Postoperative days 271–365**
No infection	*Reference*	*Reference*	*Reference*	*Reference*
Any infection	2.25 [2.13–2.38] *p* < 0.001	1.88 [1.74–2.04] *p* < 0.001	1.44 [1.29–1.62] *p* < 0.001	1.11 [1.00–1.28] *p* < 0.05
**Single infections**				
Sepsis	4.38 [3.90–4.93] *p* < 0.001	3.26 [2.74–3.89] *p* < 0.001	2.10 [1.61–2.73] *p* < 0.001	1.35 [0.95–1.91] *p* > 0.05
Pneumonia	2.60 [2.37–2.85] *p* < 0.001	2.19 [1.92–2.50] *p* < 0.001	1.70 [1.40–2.06] *p* < 0.001	1.32 [1.02–1.70] *p* < 0.05
Urinary tract infection (UTI)	1.26 [1.05–1.52] *p* < 0.05	1.21 [0.94–1.56] *p* > 0.05	1.13 [0.79–1.63] *p* > 0.05	1.07 [0.67–1.69] *p* > 0.05
Surgical site infection (SSI)	1.16 [1.04–1.30] *p* < 0.05	1.09 [0.93–1.27] *p* > 0.05	0.98 [0.79–1.22] *p* > 0.05	0.89 [0.67–1.17] *p* > 0.05
**Multiple infections**				
Sepsis combined with pneumonia, UTI, or SSI	5.35 [4.62–6.19] *p* < 0.001	3.69 [2.82–4.41] *p* < 0.001	2.12 [1.51–2.98] *p* < 0.001	1.22 [0.77–1.92] *p* > 0.05
Pneumonia combined with UTI or SSI	3.15 [2.40–4.13] *p* < 0.001	2.51 [1.69–3.74] *p* < 0.001	1.79 [1.00–3.22] *p* = 0.05	1.27 [0.59–2.77] *p* > 0.05
UTI combined with SSI	0.49 [0.16–1.43] *p* > 0.05	0.66 [0.16–2.68] *p* > 0.05	1.06 [0.16–6.93] *p* > 0.05	1.69 [0.16–17.89] *p* > 0.05

* Cox regression models were adjusted for age, sex, surgery-specific co-variables (procedure type, elective/emergent surgery, reoperation, year of surgery, perioperative GI cancer diagnosis, hospital area, postoperative length of stay) and patient comorbidities

^a^ CI: Confidence interval

Since any postoperative infection as a predictor violated the proportional hazards assumption (Supplementary Fig. 1), time-specific hazard ratios for mortality were estimated by the addition of an exposure-time interaction term to the Cox models. Time-specific adjusted HRs for one-year mortality from postoperative days 30, 91, 181, and 271 until end of follow-up (postoperative day 365) were 2.25 (95% CI: 2.13–2.38;*p* < 0.001), 1.88 (1.74–2.04;*p* < 0.001), 1.44 (1.29–1.62;*p* < 0.001), and 1.11 (1.00–1.28;*p* < 0.05) for patients with any postoperative infection compared with patients with no postoperative infection (*p*_time−interaction_<0.001). Time-specific adjusted HRs for mortality from postoperative days 30 and 271 until end of follow-up were 4.38 (3.90–4.93;*p* < 0.001) and 1.35 (0.95–1.91; *p* > 0.05) for sepsis, 2.60 (2.37–2.85;*p* < 0.001) and 1.32 (1.02–1.70;*p* < 0.05) for pneumonia, 1.26 (1.05–1.52;*p* < 0.05) and 1.07 (0.67–1.69;*p* > 0.05) for UTI, and 1.16 (1.04–1.30;*p* < 0.05) and 0.89 (0.67–1.17;*p* > 0.05) for SSI (Fig. 3; Table 3). Time-specific adjusted HRs for mortality from postoperative days 30 and 271 until end of follow-up were 5.35 (4.62–6.19;*p* < 0.001) and 1.22 (0.77–1.92;*p* > 0.05) for “sepsis combined with pneumonia, UTI, or SSI”, 3.15 (2.40–4.13;*p* < 0.001) and 1.27 (0.59–2.77;*p* > 0.05) for “pneumonia combined with UTI or SSI”, 0.49 (0.16–1.43;*p* > 0.05) and 1.69 (0.16–17.89;*p* > 0.05) for “UTI combined with SSI” (Fig. 3; Table 3). Adjusted hazard ratios for mortality from postoperative day 30 until end of follow-up for all covariables included for adjustment are presented in Supplementary Table 3.

Sensitivity analyses that investigated the association of 30-day postoperative infections and mortality over the first postoperative year (a) by excluding patients with an infection diagnosis on the day of admission, (b) with Cox regression stratified by hospital to account for the clustering of patients at the hospital level, and (c) without adjusting the regression model for *postoperative length of stay*, all showed similar results (Supplementary Table 4).

Among patients with no perioperative GI cancer diagnosis, adjusted HRs for mortality associated with postoperative infections from postoperative days 30 and 271 until end of follow-up were 2.66 (2.49–2.84;*p* < 0.001) and 1.29 (1.07–1.55;*p* < 0.05) (Supplementary Table 5). In patients with a perioperative GI cancer diagnosis, there was no association between postoperative infection exposure and mortality beyond 90 days after surgery (Supplementary Table 5). The results were similar to those presented in Fig. 3 for patients below and above 65 years of age (Supplementary Table 5). Finally, the corresponding adjusted HRs for mortality from postoperative day 30 until end of follow-up were 2.47 (2.25–2.71;*p* < 0.001) among patients undergoing non-emergent surgery and 1.91 (1.78–2.04;*p* < 0.001) among patients undergoing emergent surgery (Supplementary Table 5).

Sensitivity Cox regression analyses by different landmark times are presented in Supplementary Table 6. The use of 10- and 20-day landmark times showed a similar pattern of time-varying hazard ratios that diminished over time. For the 10-day landmark analyses, adjusted HRs for mortality associated with postoperative infections from postoperative day 10 and 270 until end of follow-up were 3.00 (2.83–3.18;*p* < 0.001) and 1.05 (0.87–1.26;*p* > 0.05). For the 20-day landmark analyses, adjusted HRs for mortality associated with postoperative infections from postoperative day 20 and 270 until end of follow-up were 2.39 (2.26–2.52;*p* < 0.001) and 1.03 (0.88–1.20;*p* < 0.005).

## Discussion

In this large nationwide registry-based study of patients undergoing gastrointestinal surgery, the hospital-verified diagnosis of any postoperative infection, as well as certain individual postoperative infection types (either alone or in combination) were associated with higher mortality risk compared with non-exposed patients over the first postoperative year. This association was more pronounced in patients who suffered from sepsis or pneumonia as opposed to UTI or SSI. However, the relative mortality risk increase associated with infections was not constant and diminished over the follow-up period. The results were robust to sensitivity and subgroup analyses. This study is the first to investigate one-year cumulative incidences of all-cause mortality and mortality rates attributed to common types of postoperative infections in a nationwide cohort of 859,766 patients undergoing gastrointestinal surgery. It is also first to demonstrate the time-varying mortality risk increase associated with postoperative infections over one year. The focus was kept on a homogenous postoperative infection profile by excluding thoracic, perianal, and purely endoscopic procedures. This choice was made to observe associations for infective complications that are most likely to be linked to the surgical event itself.

In comparison to a large study of United States veterans that investigated the associations of postoperative infections with one-year mortality following major surgery (*n* = 659,486), our study had a more balanced patient sex distribution (male gender proportion of 51% versus 91% in the US veterans’ study), slightly lower incidence of postoperative infections (2.9% versus 3.6%), and slightly higher incidence of death (5% versus 3.8%) [8]. While the relative mortality risk associated with 30-day postoperative infection was assumed to be constant over time in this study (HR = 1.89), we demonstrated that it was time-dependent and diminished with time. Furthermore, in comparison, we were able to demonstrate that the mortality risk increase varied considerably by combinations of specific postoperative infections. We included a homogenous patient group undergoing gastrointestinal surgical procedures in comparison to the US veterans’ study, which included a broad range of surgical procedures but did not investigate each surgical site (e.g. abdomen, thorax) separately.

The difference in one-year mortality risk between individual infection types could be explained by the distinct amplitude of inflammatory response caused by each infection type as well as long-term dysfunctional changes in the immune system that is proportionate with the inflammatory response [31–37]. While there are numerous reports on the long-term implications of SSIs following different surgical procedures, the incidence of other types of postoperative infections and their associations with long-term morbidity and mortality are relatively unknown [38–40]. Recent studies show that sepsis and possibly community-acquired pneumonia are associated with long-term immune dysfunction and long-term mortality [36, 37]. During the recent COVID-19 outbreak, it was reported that the severity of COVID-19 was associated with the time needed for normalization of the immune response [41]. Other authors have reported that the immune dysfunction with increased levels of proinflammatory cytokines and decreased number of T, B and NK cells persists for months after primary infection [42]. Taken together, the immune dysfunction after severe infections could explain our findings of higher relative risk of mortality within the first 90 days after surgery, and it is possible that immune parameters gradually normalize after that time point, although this should be examined further.

Since it is not possible to randomize postoperative infections in clinical trials, the observational design of this study is a valid method to examine the associations between postoperative infections and mortality. Importantly, postoperative infections are not isolated, random events; these may occur in conjunction with other complications such as pulmonary embolism, anastomotic leaks, and anesthesia-related complications. Therefore, despite that fact that we thoroughly accounted for possible confounders in our analyses, it is also possible that the observed associations between infection exposure and mortality is not causal per se, given the observational design of our study, and our results should be interpreted considering this reasoning. Nevertheless, we believe that these findings present opportunities to plan studies that target to intervene patients whose postoperative course was complicated by an event of infection. The patients can also be informed to take precautions for the intervals while they are under higher mortality risk.

## Strengths and limitations

One important strength of this study was the inclusion of patients from all ages and with equal sex distribution from the Danish general population who underwent gastrointestinal surgery, thus, our results are generalizable to the general population. For robustness, we calculated the hazard ratio estimates after testing the assumption of proportional hazards in every case and used a time-interaction term in the Cox models when this assumption was violated. Our study was not exclusive to any regions, hospitals, baseline characteristics, comorbidities or surgical modalities and is therefore representative of the general population who undergo gastrointestinal surgery in the entirety of Denmark. Therefore, there are little to no concerns about the presence of selection bias or the generalizability of our findings to similar populations. The national registries are accurate and complete for baseline characteristics such as age and sex, and we did not have any missing data regarding these. Also, since all patients were followed using Danish national registries, no patients were lost to follow-up. The procedure and infection diagnosis codes in the Danish National Patient Registry are highly accurate and complete; therefore, the risk of misclassification is estimated to be low [24, 29]. However, currently available studies that assessed the validity of infection diagnoses in the Danish National Patient Registry do not report sensitivity and specificity values, and as a result, quantitative analysis of misclassification bias was not possible [24, 29].

The most important limitation of this study is the use of a 30-day landmark time to avoid immortal time bias. The use of this landmark time frame likely resulted in the exclusion of early postoperative deaths due to postoperative infections. Although other methods such as setting postoperative infection as a time-varying exposure can be utilized to account for immortal time bias in this context, the focus of this study was the estimation of time-specific hazard ratios for postoperative infections as a predictor of mortality, and hence this variable had to be fixed over the duration of the follow-up [43].

Since this study was based on data from nationwide health registries, we did not have detailed information on the specific procedures. Nevertheless, to account for variation in procedure complexity and indication, we adjusted our regression models for various related co-variables including type of operation, open/laparoscopic surgery, emergent/elective surgery, and the preoperative diagnosis of GI cancer. Additionally, since we did not have access to data on infections or antibiotic prescriptions from the primary care sector within 30 days of surgery, we could only detect patients who had hospital-verified infections. This could have resulted in an underestimation of the number of patients who had mild SSIs or UTIs. Although there was no missing data regarding patient comorbidities, we cannot exclude the likelihood that some of these were inadequately captured. Moreover, we did not have access to quantitative measures of perioperative inflammation such as white blood cell counts or C-reactive protein (CRP) levels and therefore could not assess infection severity; although there is inconclusive evidence for associations between postoperative CRP levels and long-term survival [44, 45].

Since this is an observational study, the presence of residual confounding is inevitable despite the included adjustments. Finally, it should be noted that approximately 2.5% of deaths in the exposed group were estimated to be caused by infection; while postoperative infections were associated with an increased mortality risk, most deaths were not due to infection in the vast majority. It is possible that infection exposure is a proxy for poor general health status or a high-risk phenotype for mortality [46]. However, cause of death information in the Danish National Causes of Death Registry is largely unvalidated (by autopsy) and subject to possible misclassification [25]. Therefore, these results should be interpreted with caution.

## Conclusion

In this large nationwide registry-based study of patients who underwent gastrointestinal surgery, patients who were diagnosed with a postoperative infection had a higher relative mortality risk within the first year following the surgery, compared to those who did not have an infection. The relative mortality risk diminished over time and was more pronounced in patients who suffered from sepsis or pneumonia as opposed to less severe infections.

## Electronic supplementary material

Below is the link to the electronic supplementary material.

Supplementary Material 1

## Data Availability

Data sharing is not applicable to this study according to Statistics Denmark’s guidelines. The data used in this study is stored on Statistics Denmark’s servers. The users have signed special confidentiality and non-disclose agreements in advance and that they performed their analyses in a secure analysis environment authorized by Statistics Denmark. For more information, please check Statistics Denmark’s website: (https://www.dst.dk/Site/Dst/SingleFiles/GetArchiveFile.aspx?fi=formid&fo=dataconfidentiality--pdf&={2}).
