# Supplementary material for: Time-varying mortality risk after gastrointestinal surgery complicated by postoperative infections: a Danish Nationwide study of 859,766 patients
Source: Langenbecks Arch Surg. 2025 May 7;410(1):152. doi: 10.1007/s00423-025-03718-4 (PMC12058871; doi:10.1007/s00423-025-03718-4)
Supplement: Supplementary file 1 — Supplementary Material 1 [file 423_2025_3718_MOESM1_ESM.pdf]

## Supplementary Materials

**Title:**

Time-varying mortality risk after gastrointestinal surgery complicated by postoperative infections: A Danish Nationwide study of 859,766 patients

**Authors:**

Doruk Orgun, M.D., Ask Tybjærg Nordestgaard, M.D., Ph.D., Rasmus Peuliche Vogelsang, M.D., Ph.D., Henrik Enghusen Poulsen, M.D., D.M.Sci., Christina Ellervik, M.D., D.M.Sci., Ismail Gogenur, M.D., D.M.Sci.

**Index:**


| **Supplementary Figures and Tables** | |
|---|---|
| Supplementary Table 1 | *Page 2* |
| Supplementary Table 2 | *Page 3* |
| Supplementary Figure 1 | *Page 4* |
| Supplementary Table 3 | *Page 5-7* |
| Supplementary Table 4 | *Page 8* |
| Supplementary Table 5 | *Page 9-10* |
| Supplementary Table 6 | *Page 11* |

**Supplementary Table 1: List of International Classification of Diseases, $10^{th}$ Revision (Danish version) codes that were utilized in the study to capture postoperative infections.**

| Postoperative infection type | Danish ICD-10 codes* |
|---|---|
| **Surgical site infections** | T81.4A-T81.4C, T81.4E-T81.4J, K65, K75.0, L02.2, L03, L08, M60.8A, A48.0 |
| **Pneumonia** | J12-J18, A31.0, A48.1, T81.4P |
| **Urinary tract infections** | N10, N30.0, N30.9, T81.4U |
| **Sepsis/bloodstream infections** | A39.2, A40–A41, A02.1, A20.7, A22.7, A26.7, A32.7, A42.7, A48.3, A54.8G, A49.9A, B37.7, I33, I38-I39, J95.0A, T80.2D, T82.6, T81.4D |

**Supplementary Table 2: Causes of death within the study cohort according to 30-day postoperative infection status**

| Cause of death*, n (%) | No 30-day postoperative infection (n = 834,640) | Any 30-day postoperative infection (n = 25,126) | Total (n = 859,766) | *P* value** |
|---|---|---|---|---|
| Infection | 312 (0.7) | 85 (2.5) | 397 (0.9) | <0.001 |
| Cardiovascular | 2603 (6.6) | 262 (7.7) | 2865 (6.7) | 0.02 |
| Cancer | 27,155 (69.3) | 1673 (49.2) | 28,828 (67.7) | <0.001 |
| Pulmonary | 1302 (3.3) | 262 (7.7) | 1564 (3.7) | <0.001 |
| Gastrointestinal | 2738 (7.0) | 502 (14.8) | 3240 (7.6) | <0.001 |
| Cerebrovascular or dementia | 1416 (3.6) | 157 (4.6) | 1573 (3.7) | 0.003 |
| Urogenital | 371 (0.9) | 63 (1.9) | 434 (1.0) | <0.001 |
| Other | 3312 (8.4) | 399 (11.7) | 3711 (8.7) | <0.001 |
| Total | 39,209 | 3403 | 42,612 | |

***** Defined by the ICD-10 code that was captured as the primary cause of death in the Danish Causes of Death Registry. Corresponding codes: *Infection (A00-B99), Cardiovascular (I00-52, I70-I79), Cancer (C00-C97, D00-D09), Pulmonary (J00-J99), Gastrointestinal (K00-K93), Cerebrovascular or dementia (F03.9, I60-I69, R54), Urogenital (N00-N99)*

** P values were calculated using Pearson's chi-squared test with two-sided P values less than 0.05 were considered as statistically significant. The frequencies of cause of death types to the total number of deaths were used to populate the contingency tables.

**Supplementary Figure 1: Plot of changes in β-coefficient values between postoperative days 30 and 365 for any postoperative infection as a predictor for mortality. Scaled Schoenfield residuals over time are not shown due to Statistics Denmark's regulations.**

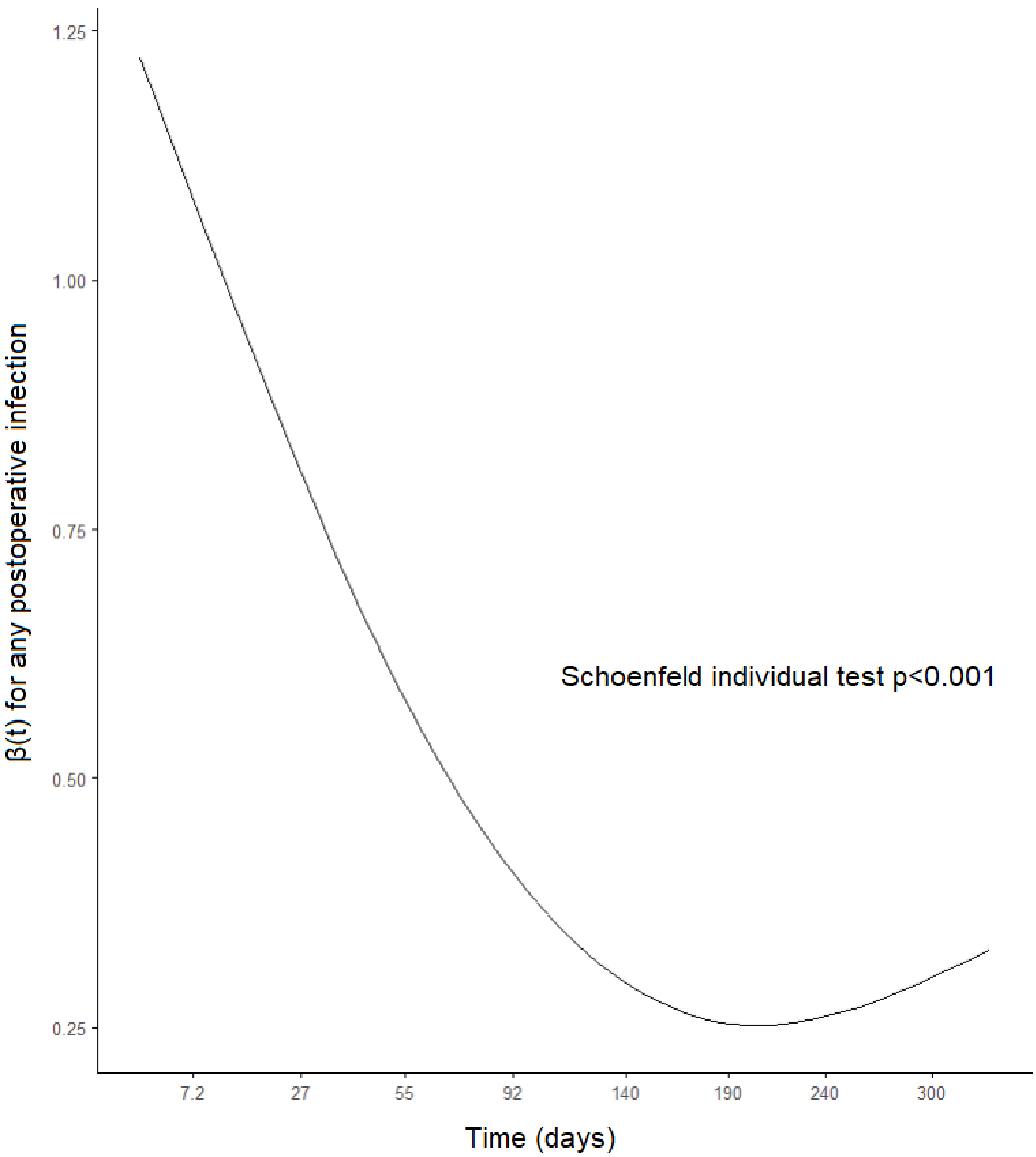

**Supplementary Table 3: Adjusted hazard ratios for mortality at postoperative day 30 for the remaining of the follow-up (until postoperative day 365) as calculated by the multivariable Cox regression model for all covariables included for adjustment.**

| **Variable** | **Adjusted hazard ratio for mortality [95% CI]** | **P-value*** |
|---|---|---|
| Any postoperative infection | | |
| No | *Reference* | |
| Yes | 2.25 [2.13 – 2.38] | <0.001 |
| Age | 1.04 [1.04 – 1.04] | <0.001 |
| Sex | | |
| Female | *Reference* | |
| Male | 1.00 [0.98 – 1.02] | <0.001 |
| Procedure type (stratification) [a] | | |
| Open abdominal wall/hernia [b] | *Reference* | |
| Laparoscopic abdominal wall/hernia | - | |
| Open lower GI | - | |
| Laparoscopic lower GI | - | |
| Open upper GI | - | |
| Laparoscopic upper GI | - | |
| Open hepato-pancreato-biliary | - | |
| Laparoscopic hepato-pancreato-biliary | - | - |
| Hospital region | | |
| Copenhagen | *Reference* | |
| Fyn | 1.07 [1.04 – 1.11] | <0.001 |
| Jutland | 0.98 [0.96 – 1.00] | 0.10 |
| Zealand | 0.98 [0.94 – 1.01] | 0.15 |
| Emergent surgery [a] | | |
| No | *Reference* | |
| Yes | - | - |

| | | |
|---|---|---|
| Reoperation | | |
| No | *Reference* | |
| Yes | 1.27 [1.19 – 1.35] | <0.001 |
| Year of surgery | | |
| 1996-2000 | *Reference* | |
| 2001-2005 | 0.89 [0.87 – 0.91] | <0.001 |
| 2006-2010 | 0.85 [0.83 – 0.88] | <0.001 |
| 2011-2015 | 0.76 [0.74 – 0.78] | <0.001 |
| 2016-2018 | 0.72 [0.69 – 0.75] | <0.001 |
| Perioperative GI cancer diagnosis [a] | | |
| No | *Reference* | |
| Yes | - | - |
| Postoperative length of stay | 1.00 [1.00 – 1.00] | |
| Diabetes | | |
| No | *Reference* | |
| Yes | 1.33 [1.30 – 1.37] | <0.001 |
| Cancer history [a] | | |
| No | *Reference* | |
| Yes | - | - |
| Coronary artery disease | | |
| No | *Reference* | |
| Yes | 1.01 [0.98 – 1.04] | 0.37 |
| Heart failure | | |
| No | *Reference* | |
| Yes | 1.45 [1.40 – 1.51] | <0.001 |
| Dialysis history | | |
| No | *Reference* | |
| Yes | 1.33 [1.30 – 1.37] | <0.001 |
| Cerebrovascular disease | | |
| No | *Reference* | |

| | | |
|---|---|---|
| Yes | 1.28 [1.23 – 1.32] | <0.001 |
| Obstructive pulmonary disease | | |
| No | *Reference* | |
| Yes | 1.43 [1.38 – 1.48] | <0.001 |
| Cirrhosis | | |
| No | *Reference* | |
| Yes | 2.93 [2.77 – 3.10] | <0.001 |

[a] Strata adjustment method was utilized for marked variables since the hazards of marked variables were not proportional over time. Thus, hazard ratios for mortality cannot be estimated for these variables.

[b] Open abdominal wall/hernia group is taken as reference due to being the procedure type with the highest number of occurrences.

**Supplementary Table 4: Sensitivity analyses - adjusted hazard ratios for mortality for postoperative days 30-365, 91-365, 181-365, and 271-365 according to 30-day postoperative infection status after a) excluding patients without an infection diagnosis on the day of admission for surgery, b) stratification for hospital codes, c) excluding postoperative length of stay variable for adjustment**

| **Adjusted hazard ratios for mortality* [95% CI]** | | | | |
|---|---|---|---|---|
| | **Postoperative days 30-365** | **Postoperative days 91-365** | **Postoperative days 181-365** | **Postoperative days 271-365** |
| No infection | *Reference* | *Reference* | *Reference* | *Reference* |
| **Main cohort (all patients)** | | | | |
| Any infection | 2.25 [2.13 – 2.38] $p<0.001$ | 1.88 [1.74 – 2.04] $p<0.001$ | 1.44 [1.29 – 1.62] $p<0.001$ | 1.11 [1.00 – 1.28] $p<0.05$ |
| **Patients without an infection diagnosis on the day of admission** | | | | |
| Any infection | 2.25 [2.13 – 2.38] $p<0.001$ | 1.89 [1.74 – 2.05] $p<0.001$ | 1.45 [1.29 – 1.64] $p<0.001$ | 1.16 [1.01 – 1.35] $p<0.05$ |
| **Patients stratified for hospital** | | | | |
| Any infection | 2.35 [2.20 – 2.51] $p<0.001$ | 1.98 [1.80 – 2.17] $p<0.001$ | 1.52 [1.33 – 1.74] $p<0.001$ | 1.18 [1.02 – 1.40] $p<0.05$ |
| **Without adjustment for postoperative length of stay** | | | | |
| Any infection | 2.25 [2.13 – 2.38] $p<0.001$ | 1.89 [1.75 – 2.04] $p<0.001$ | 1.45 [1.30 – 1.63] $p<0.001$ | 1.12 [1.00 – 1.30] $p<0.05$ |

* Cox regression models were adjusted for age, sex, surgery-specific co-variables (procedure type, elective/emergent surgery, reoperation, year of surgery, perioperative GI cancer diagnosis, hospital area, postoperative length of stay) and patient comorbidities. CI: confidence interval.

**Supplementary Table 5: Subgroup time-specific adjusted hazard ratios for mortality for postoperative days 30-365, 91-365, 181-365, and 271-365 according to 30-day postoperative infection status**

| **Adjusted hazard ratios for mortality * [95% CI]** | | | | |
|---|---|---|---|---|
| | **Postoperative days 30-365** | **Postoperative days 91-365** | **Postoperative days 181-365** | **Postoperative days 271-365** |
| **Main cohort (all patients)** | | | | |
| No infection | *Reference* | *Reference* | *Reference* | *Reference* |
| Any infection | 2.25 [2.13 – 2.38] $p<0.001$ | 1.88 [1.74 – 2.04] $p<0.001$ | 1.44 [1.29 – 1.62] $p<0.001$ | 1.11 [1.00 – 1.28] $p<0.05$ |
| **Perioperative GI cancer diagnosis status** | | | | |
| No infection | *Reference* | *Reference* | *Reference* | *Reference* |
| Any infection, no perioperative GI cancer diagnosis | 2.66 [2.49 – 2.84] $p<0.001$ | 2.22 [2.02 – 2.44] $p<0.001$ | 1.69 [1.47 – 1.94] $p<0.001$ | 1.29 [1.07 – 1.55] $p<0.05$ |
| Any infection, perioperative GI cancer diagnosis | 1.54 [1.39 – 1.70] $p<0.001$ | 1.35 [1.18 – 1.55] $p<0.001$ | 1.11 [0.91 – 1.35] $p>0.05$ | 0.91 [0.70 – 1.18] $p>0.05$ |
| **Age group** | | | | |
| No infection | *Reference* | *Reference* | *Reference* | *Reference* |
| Any infection, age <65 | 2.19 [1.96 – 2.44] $p<0.001$ | 1.86 [1.60 – 2.16] $p<0.001$ | 1.46 [1.17 – 1.81] $p<0.001$ | 1.14 [0.86 – 1.52] $p>0.05$ |
| Any infection, age ≥65 | 2.27 [2.13 – 2.42] $p<0.001$ | 1.89 [1.72 – 2.07] $p<0.001$ | 1.44 [1.26 – 1.64] $p<0.001$ | 1.05 [0.88 – 1.25] $p>0.05$ |

| | | | | |
|---|---|---|---|---|
| **Emergent surgery or non-emergent surgery** | | | | |
| No infection | *Reference* | *Reference* | *Reference* | *Reference* |
| Any infection, emergent surgery | 1.91 [1.78 – 2.04] $p<0.001$ | 1.64 [1.46 – 1.81] $p<0.001$ | 1.30 [1.13 – 1.50] $p<0.001$ | 1.04 [0.86 – 1.25] $p>0.05$ |
| Any infection, non-emergent surgery | 2.47 [2.25 – 2.71] $p<0.001$ | 2.08 [1.83 – 2.37] $p<0.001$ | 1.61 [1.34 – 1.93] $p<0.001$ | 1.24 [0.98 – 1.57] $p>0.05$ |

* Cox regression models were adjusted for age, sex, surgery-specific co-variables (procedure type, elective/emergent surgery, reoperation, year of surgery, perioperative GI cancer diagnosis, hospital area, postoperative length of stay) and patient comorbidities. CI: confidence interval.

**Supplementary Table 6: Sensitivity Cox regression analyses that utilized landmark times of 10 and 20 days.**

| **Adjusted hazard ratios for mortality * [95% CI]** | | | | |
|---|---|---|---|---|
| **Main analysis (Landmark analysis with exclusion of patients who died in the first 30 postoperative days)** | | | | |
| | **Postoperative days 30-365** | **Postoperative days 91-365** | **Postoperative days 181-365** | **Postoperative days 271-365** |
| No infection | *Reference* | *Reference* | *Reference* | *Reference* |
| Any infection | 2.25 [2.13 – 2.38] $p<0.001$ | 1.88 [1.74 – 2.04] $p<0.001$ | 1.44 [1.29 – 1.62] $p<0.001$ | 1.11 [1.00 – 1.28] $p<0.05$ |
| **Landmark analysis with exclusion of patients who died in the first 20 postoperative days** | | | | |
| | **Postoperative days 20-365** | **Postoperative days 91-365** | **Postoperative days 181-365** | **Postoperative days 271-365** |
| No infection | *Reference* | *Reference* | *Reference* | *Reference* |
| Any infection | 2.39 [2.26 – 2.52] $p<0.001$ | 1.89 [1.74 – 2.05] $p<0.001$ | 1.40 [1.24 – 1.57] $p<0.001$ | 1.03 [0.88 – 1.20] $p>0.05$ |
| **Landmark analysis with exclusion of patients who died in the first 10 postoperative days** | | | | |
| | **Postoperative days 10-365** | **Postoperative days 91-365** | **Postoperative days 181-365** | **Postoperative days 271-365** |
| No infection | *Reference* | *Reference* | *Reference* | *Reference* |
| Any infection | 3.00 [2.83 – 3.18] $p<0.001$ | 2.18 [1.98 – 2.39] $p<0.001$ | 1.51 [1.31 – 1.74] $p<0.001$ | 1.05 [0.87 – 1.26] $p>0.05$ |

* Cox regression models were adjusted for age, sex, surgery-specific co-variables (procedure type, elective/emergent surgery, reoperation, year of surgery, perioperative GI cancer diagnosis, hospital area, postoperative length of stay) and patient comorbidities. CI: confidence interval.
